# An MRI study on the relations between muscle atrophy, shoulder function and glenohumeral deformity in shoulders of children with obstetric brachial plexus injury

**DOI:** 10.1186/1749-7221-4-5

**Published:** 2009-05-18

**Authors:** Valerie M van Gelein Vitringa, Ed O van Kooten, Margriet G Mullender, Mirjam H van Doorn-Loogman, Johannes A van der Sluijs

**Affiliations:** 1 Department of orthopaedic surgery, VU medical center, 1007 MB, Amsterdam, the Netherlands; 2 Department of plastic and reconstructive surgery, VU medical center, 1007 MB, Amsterdam, the Netherlands; 3 Department of rehabilitation, VU Medical Center, 1007 MB, Amsterdam, the Netherlands

## Abstract

**Background:**

A substantial number of children with an obstetric brachial plexus lesion (OBPL) will develop internal rotation adduction contractures of the shoulder, posterior humeral head subluxations and glenohumeral deformities. Their active shoulder function is generally limited and a recent study showed that their shoulder muscles were atrophic. This study focuses on the role of shoulder muscles in glenohumeral deformation and function.

**Methods:**

This is a prospective study on 24 children with unilateral OBPL, who had internal rotation contractures of the shoulder (mean age 3.3 years, range 14.7 months to 7.3 years). Using MR imaging from both shoulders the following parameters were assessed: glenoid form, glenoscapular angle, subluxation of the humeral head, thickness and segmental volume of the subscapularis, infraspinatus and deltoid muscles. Shoulder function was assessed measuring passive external rotation of the shoulder and using the Mallet score for active function. Statistical tests used are t-tests, Spearman's rho, Pearsons r and logistic regression.

**Results:**

The affected shoulders showed significantly reduced muscle sizes, increased glenoid retroversion and posterior subluxation. Mean muscle size compared to the normal side was: subscapularis 51%, infraspinatus 61% and deltoid 76%. Glenoid form was related to infraspinatus muscle atrophy. Subluxation was related to both infraspinatus and subscapularis atrophy. There was no relation between atrophy of muscles and passive external rotation. Muscle atrophy was not related to the Mallet score or its dimensions.

**Conclusion:**

Muscle atrophy was more severe in the subscapularis muscle than in infraspinatus and deltoid. As the muscle ratios are not related to passive external rotation nor to active function of the shoulder, there must be other muscle properties influencing shoulder function.

## Background

The incidence of obstetric Brachial Plexus Lesion (OBPL) is 0.42–5.1 in 1000 live births [1,2]. Although 80–90% of the babies recover spontaneously, in 10–20% recovery is incomplete and upper limb functions do not develop normally. A substantial number of children with an OBPL will develop shoulder abnormalities consisting of contractures and/or skeletal deformities [2-6]. The typical abnormalities are internal rotation adduction contracture, posterior humeral head subluxation and deformities of humeral head and glenoid. A conventional theory proposes that these abnormalities are caused by muscle imbalance, consisting of relatively strong internal rotators and weak external rotators (see for review [5]). Yet data on shoulder muscles in OBPL children are scarce. It was shown that in OBPL children with a mean age of 7.7 years, both skeletal deformities and passive external rotation are related to infraspinatus and subscapularis muscle atrophy [7]. Another study found that in OBPL children subscapularis muscle fibres showed a decreased sarcomere length and an increased mechanical stiffness [8]. Since glenohumeral deformations arise in infancy [9], information on the relation and interaction between muscles characteristics and deformation in younger children might clarify the mechanism leading to these deformations. Besides their role in deformations, another interesting, and to our knowledge not previously explored, aspect is how limited active function of the shoulder in OBPL is related to shoulder muscle size.

Such information may be clinically relevant since it is on these muscles that treatment in OBPL infants and young children to correct deformities and improve function is often focussed.

We therefore performed a prospective study in OBPL children between 1.2 and 7.3 years to assess the relations between muscle atrophy, glenohumeral deformity and passive and active shoulder function in OBPL. The active function was assessed using the Mallet score, which was originally introduced for evaluation of shoulder outcome after neurosurgical treatment of OBPL[10] and is now widely used for evaluation of shoulder function in OBPL[11].

Generally the term atrophy is defined as reduction of size. In OBPL it is unclear whether the smaller muscle size is caused by size reduction or lack of growth, in which case hypotropic development would be a more appropriate concept. Irrespective of the mechanism the reduction of muscle size compared to the contralateral side will be referred to as atrophy.

We focussed on the infraspinatus and subscapularis muscles, but also on the deltoid muscle, since this muscle is also innervated by nerves from the brachial plexus and to our knowledge has not been studied in detail.

## Methods

### Patients

In this prospective study were included children with unilateral OBPL, Narakas classes I to III (i.e. C5-6, C5-6-7 and C5-6-7-8 lesions)[12], who had internal rotation contractures of the shoulder for which orthopaedic surgery was considered. They were analysed using MRI. Patients with neurosurgery within 12 months before MRI, or with previous shoulder surgery were excluded. Included children were scored for prior neurosurgery more than 12 months before MRI or no prior neurosurgery. They were assessed between 1998 and 2003.

The children underwent MR imaging, the younger children while being sedated. Their position was standardized with both hands on the belly. The shoulders were visualized with a three-dimensional fast imaging with steady-state precession pulse-acquisition sequense imager (TR 25 msec, TE 10 msec, flip angle 40°). The partitions used ranged from 0.8 to 3.0 mm. The protocol included imaging of both affected and normal shoulder to enable comparison with the normal anatomy. Software from Centricity RA 600(General Electric health care, Slough, United Kingdom) was used to measure angles, length and area in the MRI images. Parameters assessed focused on 1. shoulder muscles size, 2. shoulder function and 3. glenohumeral deformity.

### Shoulder muscles

In both normal and affected shoulder, muscle atrophy was measured using two methods: 1) measurements of maximum thickness of the infraspinatus and subscapularis muscle and 2) measurements of volume of a standardized segment of the subscapularis, infraspinatus and deltoid muscle. Measurements were made on transversal MR images of the shoulder regions at levels where the infraspinatus and subscapularis are shown approximately parallel to the muscle fibre direction [13].

We measured the greatest thickness of the infraspinatus and subscapularis muscle according to Pöyhiä et al. [7], to enable comparison with that study and with volume measurements. Maximum muscle thickness was measured perpendicular to the muscle direction.

**Figure 1:**
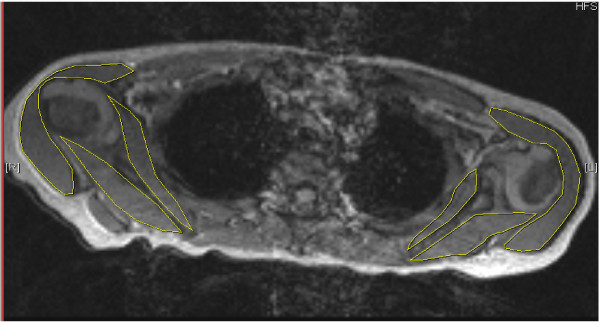
**FISP acquisition MRI in axial plane showing affected and normal contralateral shoulder**. In the affected left [L] shoulder there is a biconcave glenoid form (type 3) and humeral head subluxation. The contralateral [R] shoulder is normal. Measured areas of infraspinatus, subscapularis and deltoid are outlined.

**Figure 2:**
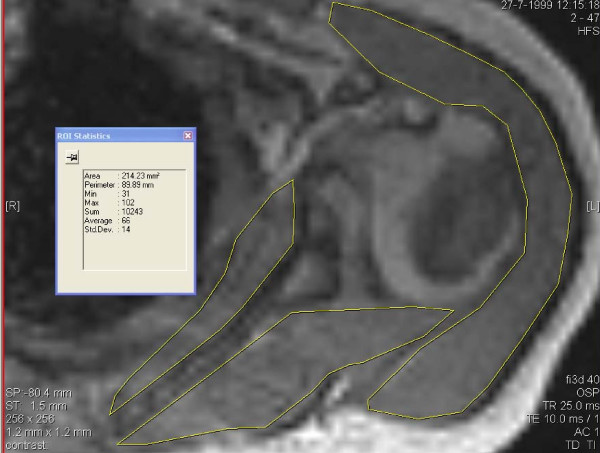
**Transversal MR image of affected shoulder with area of 3 muscles outlined and showing Centricity 600 results of area measurement of subscapularis**.

Volume measurement were performed for the infraspinatus, the subscapularis and the deltoid muscles by measuring the area of these muscles in three transversal images which is approximately parallel to the muscle fibre direction of the first two muscles. Areas were outlined manually, segmentation software was not used. (Figures 1, 2)

**Figure 3:**
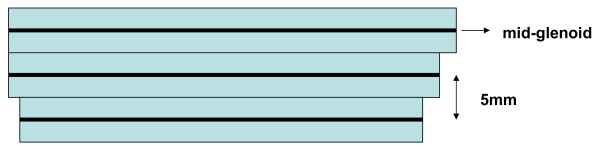
**Schematic representation of the three levels measured**. First level mid glenoid, second and third each 5 mm in caudal direction. Area multiplied by 5 mm results in volume of section. Three volume sections added is segmental volume.

It was standardized by measuring the area on the image with maximum glenoid diameter and on images 5 mm and 10 mm in caudal direction. We observed that usually at these levels areas of the muscles were maximal. Area and height were used to estimate volume. (Figure 3) Note that this is the volume of a (standardized) 15 mm transversal segment of the muscle and not the volume of the entire muscle. The calculated segmental muscle volume will be further referred to as volume.

To correct for age and inter-individual differences for each muscle the volume (or thickness) of the affected side was expressed as percentage of the volume (or thickness) of the normal side.

### Shoulder function

As a measure for the internal rotation contracture passive external rotation was measured with the shoulder in 0° abduction during outpatient assessment. Normal external rotation is 90°.

For active shoulder function the Mallet score was used[10]. Abduction, external rotation, movement of hand to neck, hand to lower spine and hand to mouth are the five dimensions of this test (Table 1). Each dimension is graded on a 5-point scale which makes the maximum Mallet score 25 points.

**Table 1:** Measurement of active shoulder function according to Mallet.

Functional parameter	Class 1	Class 2	Class 3	Class 4	Class 5
Abduction	None	<30°	30°–90°	>90°	Normal
External rotation	None	<0°	0°–20°	>20°	Normal
Hand to neck	None	Not possible	Difficult	Easy	Normal
Hand to back	None	Not possible	S1	Th12	Normal
Hand to mouth	None	Marked trumpet sign*	Partial trumpet sign*	<40° abduction	Normal

Normal function in every dimension is five points, absent function 1 point [10].

*Trumpet sign is abduction of the shoulder with simultaneous flexion of the elbow.

### Glenohumeral deformity

The glenoid form was classified according to the system proposed by Birch, et al[5] class 1: concave-flat, class 2: convex and class 3: biconcave.

**Figure 4:**
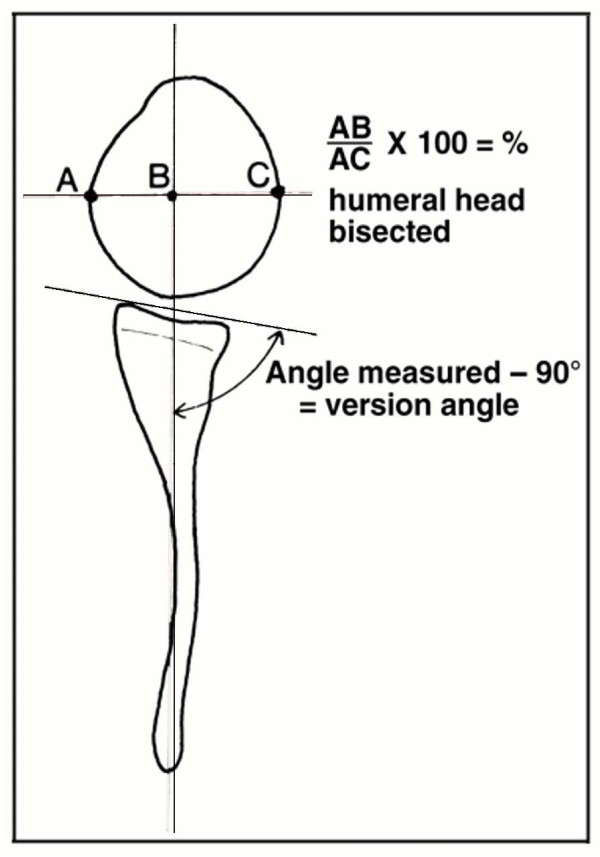
**Schematic drawing showing the method of measuring the glenoid scapular angle (GSA) and humeral head subluxation**. The glenoid scapular angle measurement (GSA) is according to Friedman et al. [14] and the humeral head subluxation according to Waters et al. [6]. For the GSA the angle in the posterior quadrant is measured and 90° are subtracted from this angle to determine glenoid version. For the subluxation the percentage of the humeral head anterior to the line from the medial margin of the scapula through the mid point of the glenoid is used. (Figure from Waters PM, Smith GR, Jaramillo D: Glenohumeral deformity secondary to brachial plexus birth palsy. *J Bone Joint Surg Am *1998, 80: 668–677. Reprinted with permission from The Journal of Bone and Joint Surgery, Inc).

Glenoid version was determined according to Friedman et al. [14], by measuring the glenoscapular angle (GSA) (Figure 4). One line was drawn from the medial margin of the scapula to the mid point of the glenoid. A second line was drawn from the anterior to the posterior margin of the cartilaginous glenoid. GSA is the angle between the medial scapula line and the posterior glenoid line, subtracted by 90°. After subtraction GSA is negative for retroversion and positive for anteversion. A GSA value around 0° was considered to be normal.

Posterior subluxation of the humeral head (further referred to as subluxation) was measured according to Waters et al.(Figure 4) [6]. The first line of the GSA measurement (scapula medial margin to midpoint glenoid) was used to measure the percentage of humeral head anterior to the middle of the glenoid fossa. The largest diameter of the humeral head was measured perpendicular to this line (AC). The anterior part of this line (AB) was divided by its total length (AC) and multiplied by 100. The normal value for this variable is approximately 50%[6]

### Statistics

All data were collected and analysed in SPSS for Windows (version 15.0). Results are given as mean +/- SD. Statistical significance of the correlations between variables was tested using either Spearman's rho in ranked variables or Pearsons r in scaled variables. Using r the coefficient of determination (r^2^) was calculated. To assess differences in muscle ratios between severe (<30%) and moderate to no subluxation (>30%) logistic regression was used. Differences between muscle ratios and differences in GSA and subluxation between normal and affected sides were assessed using t-tests. P < 0.05 was considered to be significant and all analyses were two-tailed.

## Results

In this prospective study 24 children with unilateral OBPL were included with a mean age of 3.25 years (range 14.7 months to 7.3 years), 14 girls and 10 boys. In 8 of the 24 children the affected side was left, in 16 right. Narakas classes were divided as follows: class I; 15, class II; 6 and class III; 3. Eleven children had prior neurosurgery and thirteen not. There were no complications related to the MR imaging protocol.

### Shoulder muscles

On the affected side muscle masses where usually lower than on the normal side. The mean affected/normal volume ratios for the different muscles (Table 2) are in ascending order: subscapularis muscle 50.7% ± 14.9% (range 20.8% to 77.7%), infraspinatus muscle 61.4% ± 18.0% (range 34.7% to 106.3%) and deltoid muscle 76.3% ± 14.8% (range 51.2% to 110.3%). The differences between subscapularis, infraspinatus and deltoid muscle ratios were significant (p < 0.01). Volume ratios of the three muscles were not interrelated nor were volume ratios related to Narakas class.

**Table 2:** Segmental volume and thickness ratios in subscapularis, infraspinatus and deltoid muscle of the affected shoulder.

	Subscapularis muscle		Infraspinatus muscle		Deltoid muscle	
	Mean	Range	Mean	Range	Mean	Range
Volume Ratio	50.7% ± 14.9%*	20.8% to 77.7%	61.4% ± 18.0%*	34.7% to 106.3%	76.3% ± 14.8%*	51.2% to 110.3%
Thickness Ratio	62.0% ± 16.0%**	28.3% to 87.5%	70.7% ± 17.1%**	45.0% to 106.7%	-	-

Values are given as mean ± SD with their range. * Differences between subscapularis, infraspinatus and deltoid volume ratios was significant: p < 0.01. ** Difference between subscapularis and infraspinatus thickness ratios was significant: p < 0.05.

The mean ratios for the thickness were less affected than the volume ratios. The mean ratio for the subscapularis muscle resp. infraspinatus muscle is: 62.0% ± 16.0% (range 28.3% to 87.5%) versus 70.7% ± 17.1% (range 45.0% to 106.7%). As expected muscle thickness and volume ratios of subscapularis resp infraspinatus muscle were highly related (r^2 ^= 0.466, p < 0.001 resp r^2 ^= 0.468, p < 0.001).

**Figure 5:**
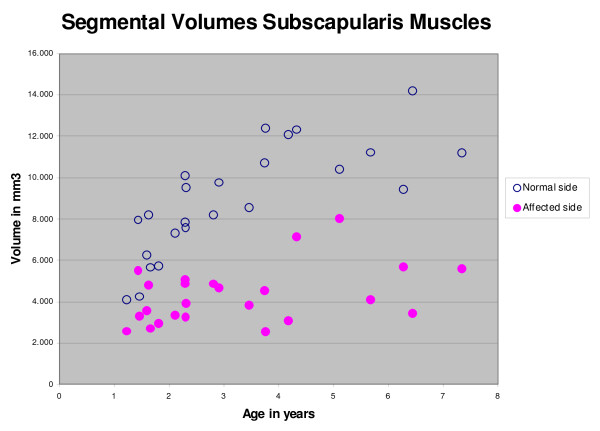
**The relation between the segmental volume of subscapularis and age**. Both normal and the affected side are shown. Significant differences between normal and affected side are found and nonaffected volume is significantly related to age.

On the normal side both volumes and thicknessess of the three muscles increased significantly with increasing age (with coefficients of determination between 0.300 and 0.579). On the affected side there was no significant increase in volume of the subscapularis muscle with increasing age (Figure 5) (p = 0.054). The affected infraspinatus and deltoid muscles did show a significant increase with age (r^2 ^= 0.362, p = 0.002 resp r^2 ^= 0.503, p < 0.001).

There were three patients with a volume muscle ratio over 100% for one of the three muscles, which means that the volumes of these muscles on the affected side were greater than on the normal side.

In children with prior neurosurgery deltoid muscle volume ratios were significantly lower than in children without surgery (r^2 ^= 0.343, p = 0.003). For infraspinatus and subscapularis ratios no significant difference was found between these groups.

### Shoulder function

Passive external rotation was less than normal (90°) in all affected shoulders with a mean of 10.6° ± 24.6° (range -50° to 60°).

The mean Mallet score in the study group was 13.3 ± 3.3 points (range 7 to 19). The sub scores for active abduction were the best (mean 3.4) and for active external rotation were worst (mean 1.6). With increasing age the Mallet score was significantly higher (r^2 ^= 0.487, p < 0.001).

Passive external rotation was related to the total Mallet score (r^2 ^= 0.245, p = 0.014).

### Glenohumeral deformity

Of the 24 affected shoulders 5 had normal type 1 glenoid form, 8 had type 2 form and 11 had type 3 form. There was no relation between age and the class of glenoid form.

The mean GSA on the affected side was significantly more retroverted compared to the normal side (Table 3): affected side -28.3° ± 15.1° (range -57° to -8°) and normal side -3.7° ± 4.2° (range -12° to 2°)(p < 0.001). The GSA was not related to age.

**Table 3:** GSA and subluxation measurements on both the affected and the normal shoulder.

	Affected side		Normal side	
	Mean	Range	Mean	Range
GSA	-28.3° ± 15.1°*	-57° to -8°	-3.7° ± 4.2°	-12° to 2°
Subluxation	30.0% ± 17.5% *	-7.4% to 51.9%	57.3% ± 7.4%	42.3% to 71.4%

Values are given as mean ± SD with their range. * Difference between the normal and affected side was significant: p < 0.001.

The mean subluxation on the affected side was significantly larger compared to the normal side: affected side 30.0% ± 17.5% (range -7.4% to 51.9%) and normal side 57.3% ± 7.4% (range 42.3% to 71.4%)(p < 0.001). Subluxation did not increase significantly with age.

In the affected shoulders the 3 different parameters of glenohumeral deformity (glenoid form, GSA and humeral subluxation) were interrelated with r^2 ^between 0.386 and 0.789 (p ≤ 0.001).

### Correlations between muscles, glenohumeral deformation and function

Glenoid form was negatively related to infraspinatus muscle volume ratio (r^2 ^= 0.493, p < 0.001), but not to subscapularis and deltoid muscle volume ratios. Glenoid form correlated negatively with infraspinatus thickness as well (r^2 ^= 0.235, p = 0.016) and not with subscapularis thickness.

Subluxation was related to muscle volume. The combination of a low volume infraspinatus ratio with a low volume subscapularis ratio predicts severe subluxation (<30%) (logistic regression: p = 0.026 and R^2 ^= 0.224). When using muscle thicknesses of these muscles as predictors no significance was reached (logistic regression: p = 0.383 and R^2 ^= 0.059). GSA was not related to any of the volume ratios.

There was no relation between passive external rotation and atrophy of any of the muscles. Neither did passive external rotation correlate with any of the three glenohumeral deformities. There was no relation between any of the muscle volume ratios and Mallet score or its dimensions.

## Discussion

This study concerning shoulder muscle atrophy in OBPL children shows 2 new findings related to: the pattern of atrophy, and the relation between muscle atrophy and both passive and active shoulder function.

### Pattern of atrophy

No consistent pattern of atrophy was found: the extent of atrophy of the various muscles was not significantly interrelated. Based on anatomical consideration we would expect a relation between subscapularis and deltoid muscle atrophy since both muscles are innervated by branches from the same (posterior) cord of the brachial plexus. Although the extent of atrophy of the three muscles was not interrelated, a general pattern of the extent of atrophy emerged.

Almost all muscles on the affected side showed atrophy, but atrophy was most evident in the subscapularis muscle (with almost 50% loss of volume). In contrast to the study of Pöyhiä et al. [7] which showed that (based on thickness measures) both subscapularis and infraspinatus atrophy were 69%, we found a substantial difference between subscapularis atrophy (volume reduction 50.7%, thickness reduction 62%) and infraspinatus atrophy (volume reduction 61%, thickness reduction 70%). The difference in atrophy is remarkable. Since the infraspinatus muscle is innervated by a higher nerve branch of the brachial plexus (the suprascapular nerve) than the subscapularis muscle (the subscapular nerves) and in OBPL most plexus lesions progress in a craniocaudal direction, the infraspinatus muscle is expected to be most affected by denervation. Since denervation generally causes severe atrophy [15] the infraspinatus muscle is expected to be most atrophic and not, as found in these studies, the subscapularis muscle. Furthermore growth in the affected subscapularis muscle was minimal. Whereas affected infraspinatus and deltoid muscle volumes correlated with age, subscapularis muscle volume did not increase significantly with increasing age, although significance was almost reached (p = 0.054). Growth retardation of this muscle is in line with growth retardation of the scapula. Two recent studies have shown that in OBPL shoulders the scapula was hypoplastic and scapular growth was impaired [16,17]. As the subscapularis muscle originates on the scapular fossa, reduced scapular growth could be related to reduced muscle growth. However, this relation is not present in the infraspinatus muscle, also originating on the scapula. Whereas in most children atrophy was found on the affected side, three children had muscle ratios over 1, suggesting the affected side had a greater muscle volume than the normal side. This might be explained by the paradoxal enlargement of muscles which sometimes occurs after denervation and has been described before by Petersilge et al [18].

The pattern of atrophy does not seem to match the pattern of denervation. The premise that denervation always leads to atrophy may be incorrect in young children. The effect of (partial) nerve injuries on growing muscles is unclear. Another mechanism might be operative. According to Williams et al. [19] immobilization in shortened position causes muscle atrophy.

### Hypothesis on subscapularis atrophy

We propose the following hypothesis to explain the difference in atrophy between infraspinatus and subscapularis. We suggest that the infraspinatus muscle is more affected by denervation than the subscapularis muscle. This results in a weaker infraspinatus with reduced external rotation force, which leads to a more internal rotated shoulder. The subscapularis muscle, not lengthened sufficiently by its antagonist the infraspinatus muscle, remains relatively short and immobilized. Being predominantly shortened the subscapularis muscle is more affected by the atrophy mechanism described by Williams et al. [19] than the relatively elongated infraspinatus muscle. This is in line with a recent study which suggests that secondary changes in muscle fibre properties occur as a result of long standing lack of sufficient passive stretch[8]. If correct this hypothesis would suggest that preserving passive range of motion and prevention of internal rotation contracture of the shoulder by stretching the subscapularis would be facilitated by using botulinum toxin in the subscapularis to reduce muscle force and tone in that muscle.

### Relation between muscle atrophy and passive and active function

The volumes of external rotator (infraspinatus) and internal rotator (subscapularis) muscles were not related to passive external rotation (measure of internal rotation contracture). Hence, we could not confirm the findings of progressive reduction of passive external rotation with increasing infra- and subscapularis atrophy as described by Pöyhiä et al.

*Neither were v*olume ratios of infraspinatus, subscapularis and deltoid muscles related to total Mallet score nor to one of its 5 dimensions. The absence of a relation between infraspinatus and active external rotation and particularly deltoid atrophy and Mallet score is remarkable. Other external rotators are available but one would expect that the volume of the deltoid muscle, a shoulder abductor, to be related to least 3 dimensions of the Mallet score (that is abduction, hand to neck, hand to mouth).

In this age group measuring atrophy is inadequate to predict the more complex relation between muscle size and passive and active function. Apparently in this age group other muscle factors besides size might play a role in passive and active shoulder function. Such changes in muscles have been described in the cited study which found reduced sarcomere length and greater muscle fibre stiffness in subscapularis biopsies in OBPL children[8].

### Measurement of muscle size

Muscle atrophy was measured, in transversal MR images which display infraspinatus and subscapularis parallel to the direction of the muscle fibres at the chosen level [13]. We used two separate measures: maximal thickness and volume of a standardised 15 mm high segment. In our opinion volume measurement has advantages. Muscle thickness is variable and could depend highly on the position of the muscle. An atrophic muscle for example can be thick when shortened by shoulder position, while a normal muscle can be thin when stretched. This problem can be solved by measuring the area and use these area's to calculate segmental volume. Although we outlined muscle contours manually, segmentation software could be useful in the future.

Another advantage of area and volume estimation is the ability to measure the deltoid muscle, which is also innervated by a nerve from the brachial plexus (the axillary nerve). In the transversal MR images this muscle is displayed approximately perpendicular to the fibre direction. Because of this muscle's position and the great inter-individual variation in the shape of its different heads, measuring maximal thickness is not precise and hard to standardize so using volume measurement is preferable.

The choice for the use of volume measurement is further supported by our observation is that volume is related to glenohumeral deformities, as shown by the higher correlations in this study: volume ratios of the infraspinatus and subscapularis muscle could significantly predict subluxation and glenoid deformity, but the thickness ratios of these muscles could not.

### Relation between muscle atrophy and glenohumeral deformity

Muscle atrophy was related to glenohumeral deformity. Glenoid deformity was related to severe infraspinatus atrophy and humeral subluxation was related to both more severe infraspinatus and subscapularis atrophy. This confirms the study of Pöhyia [7] in children with a mean age of 7.7 years. Although the role of muscles in the development of glenohumeral deformity in OBPL seems logical and has long been suggested, the cited study was the first to quantitatively show this relation. Our study shows that this relation is already present in younger children (mean age 3.3 years)

In our study and confirming the study performed by Kozin et al. [20] no significant relation was shown between age and glenohumeral deformities. Mean ages in these studies were 3.3 years (range 1.2 to 7.3) resp. 4.9 years (range 1.8 to 10.1). In a study on infants (mean age 5.2 months, range 2.7 to 8.7) glenohumeral deformities increased significantly with age [9]. Apparently, glenohumeral deformities arise particularly in the first period of life, the rate of deformation reducing in later years. This suggests that prevention of deformities should focus on the first year of life.

## Conclusion

There was substantial atrophy of the subscapularis, infraspinatus and deltoid muscles in OBPL children. Remarkable findings were that the atrophy of the three muscles was not interrelated, that the subscapularis muscle was most severely affected and that muscle ratios were related to glenohumeral deformation but not related to passive external rotation nor to the total Mallet score and its dimensions.

## Competing interests

The authors declare that they have no competing interests.

## Authors' contributions

VMvGV and JAvdS did design, data acquisition, analysis, and writing. EOvK MM and MHVD revised the manuscript critically for important intellectual content. All approved the final version
