# An MRI study on the relations between muscle atrophy, shoulder function and glenohumeral deformity in shoulders of children with obstetric brachial plexus injury

**DOI:** 10.1186/1749-7221-4-9

**Published:** 2009-07-08

**Authors:** Valerie M van Gelein Vitringa, Ed O van Kooten, Richard T Jaspers, Margriet G Mullender, Mirjam H van Doorn-Loogman, Johannes A van der Sluijs

**Affiliations:** 1 Department of Orthopaedic Surgery, VU medical center, 1007 MB, Amsterdam, The Netherlands; 2 Department of Plastic and Reconstructive Surgery, VU medical center, 1007 MB, Amsterdam, The Netherlands; 3 Research Institute MOVE, Faculty of Human Movement Science, VU University Amsterdam, 1081 BT, Amsterdam, The Netherlands; 4 Department of Rehabilitation, VU Medical Center, 1007 MB, Amsterdam, The Netherlands

## Correction

After publication of this work [1], we noted that we inadvertently failed to include the complete list of all co-authors. The full list of authors has now been added and the Authors' contributions and Competing interests sections modified accordingly.

## Competing interests

The authors declare that they have no competing interests.

## Authors' contributions

VMvGV, RTJ and JAvdS did design, data acquisition, analysis, and writing. EOvK MM and MHVD revised the manuscript critically for important intellectual content. All authors approved the final version.
